# Circadian Phase-Shifting Effects of Bright Light, Exercise, and Bright Light + Exercise

**DOI:** 10.5334/jcr.137

**Published:** 2016-02-26

**Authors:** Shawn D. Youngstedt, Christopher E. Kline, Jeffrey A. Elliott, Mark R. Zielinski, Tina M. Devlin, Teresa A. Moore

**Affiliations:** College of Nursing and Health Innovation and College of Health Solutions, Arizona State University, Phoenix, AZ, US; Phoenix VA Health Care System, Phoenix, AZ, US; Department of Health and Physical Activity, University of Pittsburgh, Pittsburgh, PA, US; Center for Circadian Biology, University of California, San Diego, CA, US; Department of Psychiatry, Harvard Medical School, VA Boston Healthcare System, Boston, MA, US; South Carolina Department of Health and Environmental Control, Columbia, SC, US; Department of Exercise Science, University of South Carolina, Columbia, SC, US

**Keywords:** humans, ultra-short sleep wake cycle, 6-sulphatoxymelatonin, additive effect, young adults

## Abstract

Limited research has compared the circadian phase-shifting effects of bright light and exercise and additive effects of these stimuli. The aim of this study was to compare the phase-delaying effects of late night bright light, late night exercise, and late evening bright light followed by early morning exercise. In a within-subjects, counterbalanced design, 6 young adults completed each of three 2.5-day protocols. Participants followed a 3-h ultra-short sleep-wake cycle, involving wakefulness in dim light for 2h, followed by attempted sleep in darkness for 1 h, repeated throughout each protocol. On night 2 of each protocol, participants received either (1) bright light alone (5,000 lux) from 2210–2340 h, (2) treadmill exercise alone from 2210–2340 h, or (3) bright light (2210–2340 h) followed by exercise from 0410–0540 h. Urine was collected every 90 min. Shifts in the 6-sulphatoxymelatonin (aMT6s) cosine acrophase from baseline to post-treatment were compared between treatments. Analyses revealed a significant additive phase-delaying effect of bright light + exercise (80.8 ± 11.6 [SD] min) compared with exercise alone (47.3 ± 21.6 min), and a similar phase delay following bright light alone (56.6 ± 15.2 min) and exercise alone administered for the same duration and at the same time of night. Thus, the data suggest that late night bright light followed by early morning exercise can have an additive circadian phase-shifting effect.

## Introduction

Under everyday conditions, the circadian system is synchronized to the earth’s 24-h rotation to promote adaptation to the environment. Synchronization occurs through exposure to daily time cues (zeitgebers). However, malsynchronization between circadian timing and environmental demands is a common condition with numerous negative sequelae. For example, shift-workers, who comprise about 20 percent of the work force [1], suffer chronic sleep disruption, and an increased risk of cancer, depression, cardiovascular, endocrine, and gastrointestinal disease, and work-related accidents [2,3,4,5]. Likewise, delayed sleep phase syndrome has been associated with sleep curtailment and a relatively high prevalence of depression [6] and obesity [7]. Conversely, improved circadian synchronization might prevent or attenuate associated health problems.

Bright light is considered the most important zeitgeber. However, bright light has limited efficacy for many blind individuals [8], and it can elicit side effects, such as eye strain and headaches, as well as mania in individuals with a history of mania [9]. Therefore, there is a need to explore alternative or adjuvant methods for shifting circadian timing.

Rodent studies [10,11,12,13] and human studies [14,15,16,17,18,19] have established that exercise can also have a significant circadian phase-shifting effect, and can facilitate entrainment to a shifted light-dark and sleep/wake schedule. Although it is generally assumed that the phase-shifting effect of exercise is far less potent than that associated with bright light, there is little empirical evidence supporting this assumption. On the contrary, we found that 1 h of moderately vigorous treadmill exercise in the late night/early morning elicited phase shifts that were approximately 1/3 of the shifts produced by 3 h of bright light (3,000 lux) [20], which was analogous to differences reported for phase-shifting effects of 1 h vs. 3 h of bright light [21].

Likewise, Van Reeth et al. [14] found approximately equivalent phase-shifting effects of 2.5 h of exercise at 50% of maximum effort, equivalent to moderate walking for most people, and 3 h of bright light of 5,000 lux, which elicits approximately 90% of the phase-shifting effects of the brightest experimental light. Thus, it is plausible that vigorous exercise and bright light of equivalent duration and timing might have similar phase-shifting effects.

In hamsters, a fascinating interaction between phase-shifting effects of light and exercise has been observed. Light and exercise antagonize each other’s phase-shifting effects when administered within a few hours of each other [22,23,24], but these stimuli can have additive or synergistic effects when separated by more than 4 hours [23, 25, 26]. Demonstration of a similar additive effect in humans could have considerable practical utility for rapidly shifting the human circadian system.

The aims of this pilot study were (1) to compare phase-delaying effects of bright light alone and exercise alone administered at the same time of night and for the same duration, and (2) to compare phase-delaying effects of these independent stimuli with that associated with bright light followed by exercise. The bright light + exercise treatment was designed to elicit additive phase-delaying effects both by separating the stimuli by > 4 h (as shown in hamsters) and by focusing both stimuli at phase delay regions of the respective phase response curves for these stimuli [20,27].

## Materials and Methods

Participants were six aerobically fit and active adults ages 18–30 y (3 women, 3 men). Demographic data are displayed in Table 1. Exclusion criteria included (1) recent shift-work experience (previous 2 months) or travel across multiple time zones (previous 2 weeks); (2) abnormal sleep-wake schedule (bedtime before 9:00 pm or after 1:00 am; wake time before 5:00 am or after 10:00 am); (3) extreme night owl or morning lark based upon the Horne-Ostberg Morningness-Eveningness Questionnaire (<31 or >69, respectively) [28]; (4) self-reported depression (Beck Depression Inventory > 16) [29]; (5) having more than one of the major risk factors for coronary artery disease; (6) and inadequate levels of aerobic exercise (i.e., exercise < 3 days per week, and/or < 20 min/day, and/or < 70% maximal effort). Each volunteer signed written informed consent approved by the University of South Carolina Institutional Review Board.

**Table 1:** Demographic information about the participants.

	Men (n = 3)	Women (n = 3)

Age (yr)	23.7 ± 3.2	20.7 ± 1.2
Height (cm)	172.7 ± 7.6	159.2 ± 5.3
Weight (kg)	66.2 ± 9.3	53.7 ± 2.6
Beck Depression Inventory	2.0 ± 1.7	1.0 ± 1.7
Horne-Ostberg	53.8 ± 9.3	59.3 ± 0.6
Morningness-Eveningness Questionnaire	Intermediate	Intermediate
Usual Sleep Duration (min)	450 ± 52.2	410.0 ± 45.8

In a within-subjects counterbalanced design, participants completed each of three 2.5-day laboratory protocols (described below). The three protocols were each separated by 1–3 weeks.

**Pre-Experimental Weeks.** During one week prior to each experimental protocol, participants maintained stable sleep-wake schedules (i.e., bed and wake times not varying by more than 1.5 h) at times that were consistent with their average bedtimes and wake times. Adherence to these schedules was verified by continuous wrist actigraphic recording and sleep diaries. Participants were also asked to maintain their usual exercise habits during the pre-experimental week, and to abstain from alcohol and caffeine consumption for the two days preceding entrance into the laboratory.

**Figure 1:**
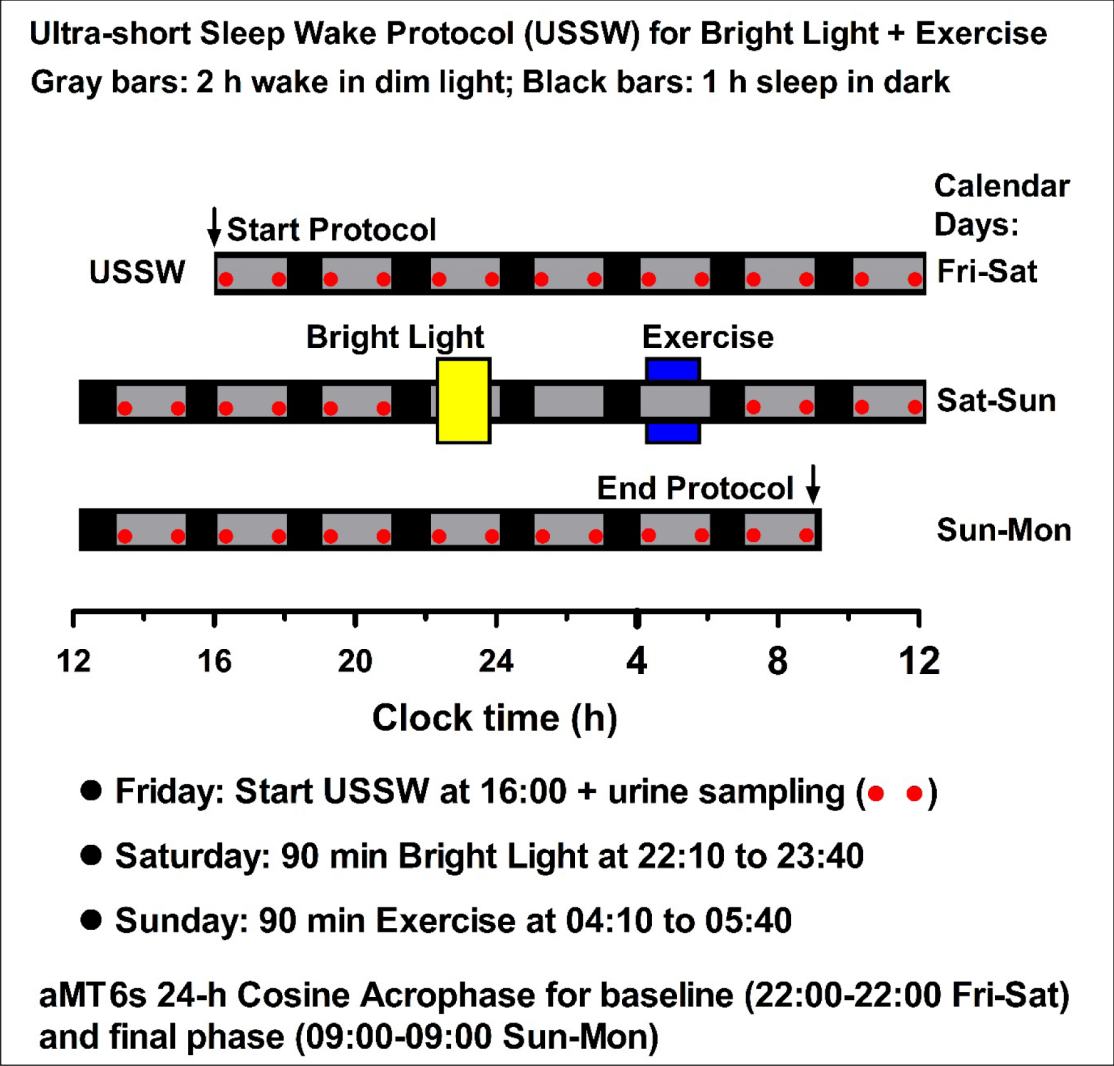
**Experimental protocol: evening bright light followed by early morning exercise.** Participants adhered to an ultra-short sleep-wake cycle beginning at 1600 h on Friday, and were subsequently exposed to 90 min of bright light (5000 lux, 2210–2340 h) followed 4.33 h later by 90 min of exercise (0410–0540 h). In the other two treatments, subjects received bright light alone or exercise alone at 2210–2340 h. Phase shifts of the aMT6s rhythm were calculated by subtracting final post treatment acrophase from baseline acrophase.

**Laboratory Protocol: Ultra-Short Sleep-Wake Cycle.** For each 2.5-day laboratory protocol, participants entered the laboratory at 1600 h on DAY 1 (Friday afternoon) and remained for 58–65 h until DAY 4 (Monday morning) (Figure 1). Upon arrival, participants were told which of the three treatments they would be performing: bright light alone, exercise alone, or bright light + exercise. Throughout each 2.5-day laboratory observation, participants followed a 3-h ultra-short sleep-wake schedule, in which participants were given 2-h intervals of out-of bed wakefulness in dim light (≤20 lux), followed by 1-h intervals for sleep in darkness (<1 lux). Chronobiologic protocols, such as the ultra-short sleep-wake cycle, are used to precisely assess circadian rhythms whose measurement can otherwise be masked by numerous environmental and behavioral factors, including, sleep, physical activity, caloric intake, posture, light exposure, and ambient temperature. By distributing these masking effects equally around the 24-h day, one can make inferences about the circadian system. Past research has shown that this schedule can be well-tolerated for up to 10 days [27, 30, 31].

Adherence to the ultra-short sleep-wake schedule was verified by 24-h observation and continuous wrist actigraphic recording (Octagonal Basic Motionlogger, Ambulatory Monitoring, Inc, Ardsley, NY). Participants spent the 2-h wake intervals as they wished, e.g., speaking with visitors or other participants or research staff, watching movies, or reading. However, napping and exercise (outside of experimental treatment) were prohibited. At min 30 of each 2-h wake period, participants consumed an isocaloric meal, standardized by a registered dietician (TAM) based on each participant’s body weight, age, gender, and physical activity level [32]. Water and non-caffeinated, calorie-free, nonalcoholic drinks were available ad libitum.

For the 1-h sleep intervals, participants remained recumbent in darkness and were asked to attempt to sleep. Immediately after the second sleep interval (at 10:00 pm), resting recumbent heart rate was established with a heart rate monitor (FS1, Polar Electro Inc., Lake Success, New York) and used for subsequent standardization of the intensity of the exercise treatments.

**Experimental Treatments.** Following a baseline period of approximately 30 h, participants completed one of the three counterbalanced treatments, beginning on the evening of DAY 2: **Bright Light Alone, Exercise Alone, or Bright Light + Exercise** (Figure 1).

**Bright Light Alone.** Participants were exposed to 5,000 lux light for 90 min from 2210–2340 h. The treatment was administered by a panel of light boxes (UV protected) situated 1.5 m from the field of vision (Brite Lite, Apollo Light Systems, Inc., American Fork, Utah). Light exposure at eye level was verified by periodic sampling with a calibrated light meter.

**Exercise Alone.** Participants exercised on a treadmill for 90 min from 2210–2340 h. According to our previous research, the exercise and bright light occurred at similarly sensitive phase delay regions of the respective phase response curves for exercise and bright light (20, 27). The exercise involved 20-min intervals at 65-75% heart rate reserve (HRR), interspersed with 5- min recovery intervals at 30–40% HRR. Thus, total durations at 65–75% and 30–40% HRR were 75 min and 15 min, respectively. The exercise intensity for each participant was determined from age-predicted maximal heart rate (i.e., 220-age) and his/her resting heart rate. Exercise intensity was maintained using a Polar heart rate monitor, and necessary adjustments to speed and/or slope of the treadmill were made. Each participant spent ≥ 90% of his/her time in the prescribed intensity range.

**Bright Light + Exercise.** Participants were exposed to bright light (5,000 lux) at 2210–2340 h on DAY 2, followed 4.33 h later on DAY 3 by exercise at 0410–0540 h (20-min intervals at 65%–75% HRR, with 5-min recovery intervals at 30–40% HRR.

**Experimental Measurement.** Urine samples were collected every 90 min; i.e., twice during every wake period: 0–15 min after arising and 15–30 min before bedtime, as well as following any other voidings. The time and volume of each sample were recorded and the sample was frozen (–80 ^o^C). The samples were subsequently shipped to the Emory University Yerkes Primate Center (Atlanta, GA) and assayed (via radioimmunooassay) for 6-sulphatoxymelatonin (aMT6s), the primary urinary metabolite of melatonin.

**Circadian phase and assessment of shifts.** From the aMT6s concentration, the urine volume, and the collection times, the aMT6s excretion rate (ng/h) was computed for each collection interval (the interval between one voiding and the next one) and subsequently associated with each 5-min interval within the collection interval. From this time series of 5-min intervals, the circadian analyses were computed. Baseline circadian phase was established from aMT6s data collected during the 24 h preceding the first experimental treatment. Baseline acrophase (peak time) of aMT6s excretion was established by least-squares estimation of the best-fitting 24-h cosine curve of the 5-min data. Final aMT6s acrophase was established from aMT6s data collected during the final 24-h in the laboratory.

According to convention, the phase shift in aMT6s acrophase was determined by subtracting the final acrophase from the baseline acrophase. Phase shifts were compared between treatments via repeated measures ANOVA with planned comparisons that fit the hypotheses: i.e., Bright Light vs. Exercise, Bright Light vs. Bright Light + Exercise, and Exercise vs. Bright Light + Exercise. Based on the small sample size, an a priori decision was made to not compare other markers of circadian phase (e.g., aMT6s onset), leading to multiple comparisons and increased susceptibility to Type I error. Because of expected low power to detect statistically significant results, effect sizes were also calculated for each treatment, calculated as the difference between baseline and final aMT6s acrophase divided by the pooled standard deviation of these acrophases.

**Figure 2:**
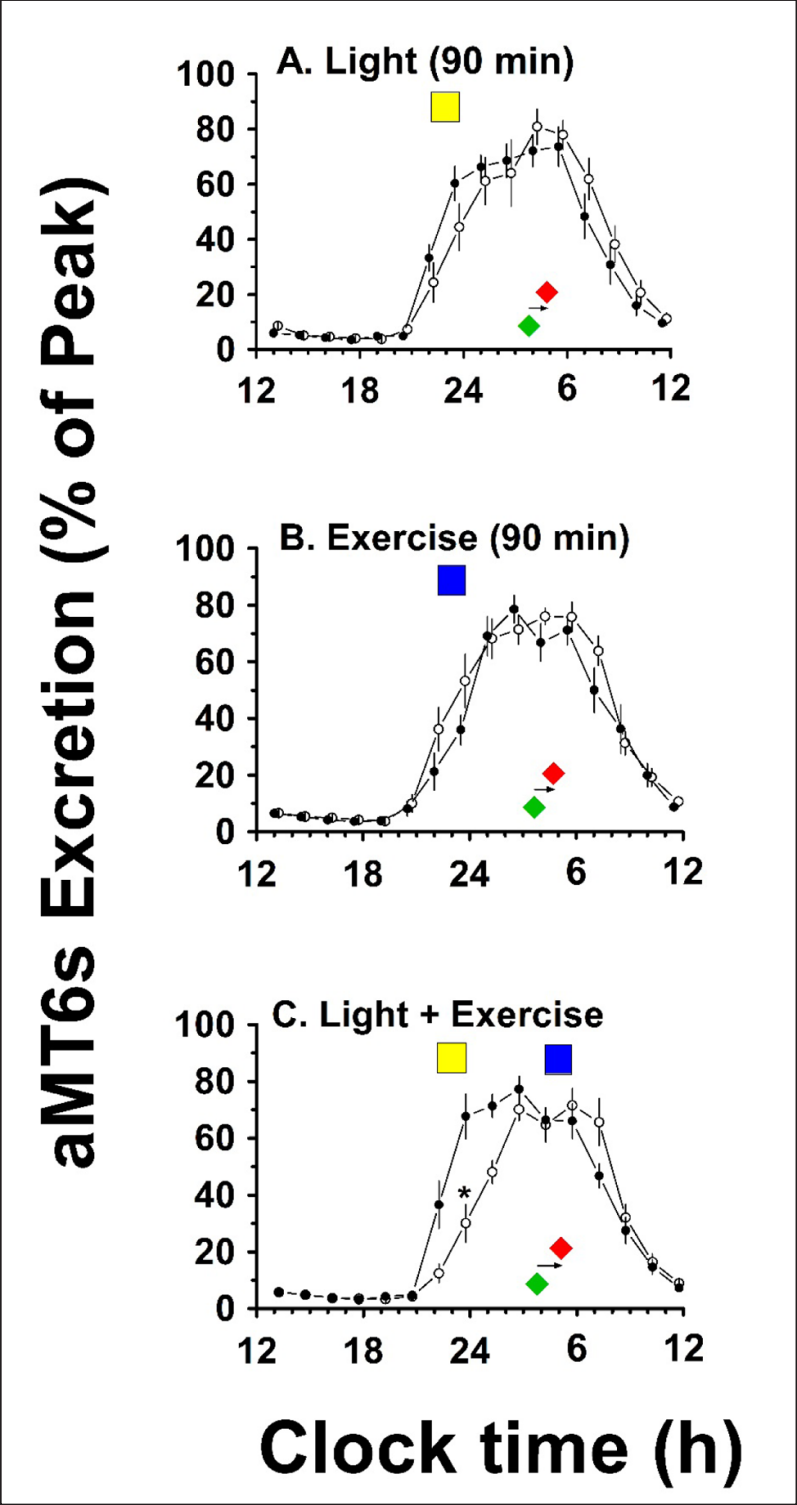
**24-h rhythms of aMT6s Excretion at baseline and after treatment.** Individual urinary 6-sulphatoxymelatonin data (aMT6s) were averaged into 90-min bins, normalized to percent of peak, and group means (+/– SEM, N = 6) plotted on a 12 noon to 12 noon axis to yield synchronized 24-h profiles representing rhythm timing and waveform at baseline (●) and post-treatment (ο). Color-filled rectangles represent the timing of light and exercise stimuli (light: yellow; exercise: blue). Color filled diamonds underneath the curves represent mean acrophase times before (green) and after (red) treatment. ANOVAs for the normalized 90-min time series (panels A,B,C) underscored robust 24-h rhythmicity [p’s < 0.001] and confirmed a significant phase shift (interaction) for the Light + Exercise treatment, but not for the other treatments.

To further explore shifts across all participants, we created 90-min bins of averaged aMT6s ng/h time series data for all subjects and normalized each time series to the peak aMT6s excretion (ng/h) (Figure 2). ANOVA was then used to explore whether there were shifts in these normalized data.

## Results

The goodness of fit data (all q ≥ 0.86) and circadian quotient (CRQ: amplitude/mesor) data (Table 2) suggest robust circadian profiles of aMT6s excretion before and after the treatments. Similarly, Figure 2 illustrates the robustness of the normalized aMT6s circadian rhythm data before and after the treatments.

**Table 2:** aMT6s baseline, timing of treatments, and phase shifts in the aMT6s acrophase for the individual participants.

Subject	Bright Light			Exercise			Bright Light + Exercise		
	Base aMT6s Acrophase	Timing: After Phase (h:min)	aMT6s Acrophase Delay (min)	Base aMT6s Acrophase	Timing: After Phase (h:min)	aMT6s Acrophase Delay (min)	Base aMT6s Acrophase	Timing After Acrophase for Light & Exercise	aMT6s Acrophase Delay (min)

01	0137 h	–2:41	–71	0244 h	–3:49	–39	0205 h	–3:10 & +2:50	–64
02	0419 h	–5:24	–79	0410 h	–5:15	–34	0440 h	–5:44 & +0:16	–74
03	0147 h	–2:52	–55	0115 h	–2.20	–89	0244 h	–3:49 & +2:71	–85
04	0443 h	–5:48	–46	0438 h	–5:43	–30	0426 h	–5:31 & +0:29	–83
05	0619 h	–7:24	–43	0538 h	–6:43	–48	0503 h	–6:08 & -0:08	–98
06	0345 h	–4:50	–45	0302 h	–4:07	–43	0313 h	–4:18 & 1:42	–81

**Figure 3:**
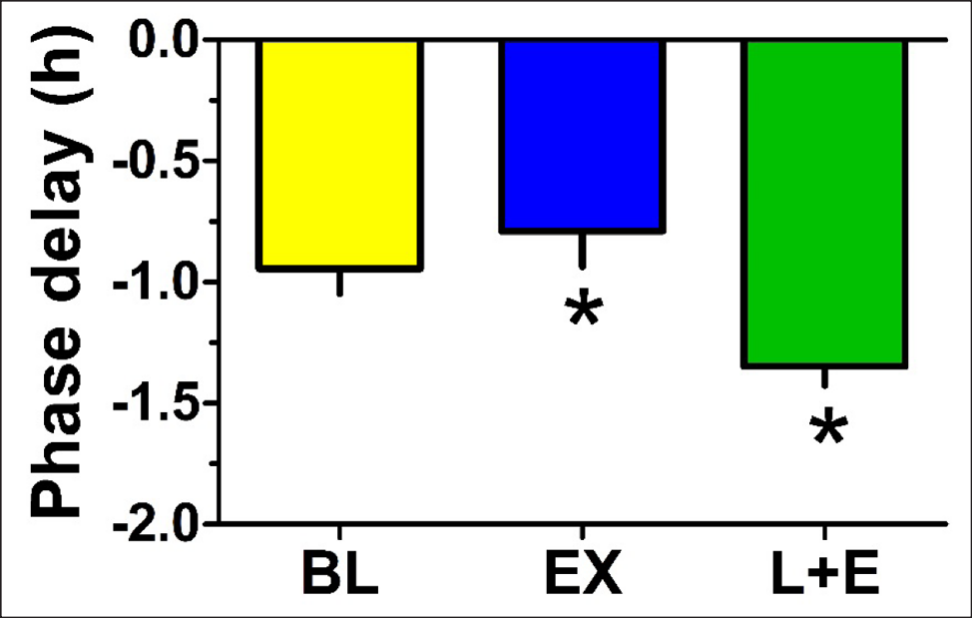
**Phase delays in aMT6s acrophase were greatest with light followed by exercise.** The bar graph contrasts the group mean (-SEM, n = 6) phase delay shifts observed in aMT6s rhythm 24-h cosine acrophases determined pre- vs. post-stimulation in the three treatments [Bright Light (BL); Exercise (EX); and Bright Light + Exercise (L+E)]. ANOVA revealed that the phase delay following L + E was significantly greater than that following EX. Other comparisons were not significant.

Phase shift data for individual participants are displayed in Table 2. Mean aMT6s acrophase phase shift data are displayed in Figure 3 and Table 3. Mean aMT6s acrophase phase shift following Bright Light Alone, Exercise Alone, and Bright Light + Exercise was 56.6 ± 15.2 min, 47.3 ± 21.6 min, and 80.8 ± 11.6 min, respectively. Corresponding effect sizes for these shifts were 0.53, 0.54, and 1.04, respectively.

**Table 3:** Mean aMT6s data across the six participants.

Treatment	Baseline aMT6s Acrophase (h)	Baseline CRQ Amplitude/Mesor	Stimulus Timing: H After Baseline Acrophase	Final aMT6s Acrophase (hr:min)	Final CRQ	aMT6s Phase Shift min

Bright Light	0345 ± 1:48	1.22 ± 0.08	–4.83 ± 1.81	0442 ± 1:43	1.29 ± 0.13	–56.6 ± 15.2
Exercise	0335 ± 1:33	1.21 ± 0.16	–4.66 ± 1.56	0422 ± 1:20	1.24 ± 0.12	–47.3 ± 21.6
Bright Light + Exercise	0342 ± 1:11	1.24 ± 0.11	Light: –4.78 ± 1.19 EX: 1.21 ± 1.19	0503 ± 1:19	1.31 ± 0.10	–80.8 ± 11.6

ANOVA revealed no significant treatment by time interaction for phase shifts following Bright Light Alone vs. Exercise Alone [F(1, 5) = 0.64, p = 0.46]. The phase shift following Bright Light + Exercise was marginally greater than that following Bright Light Alone [F(1, 5) = 5.50, p = 0.07], and significantly greater than that following Exercise Alone [F(1, 5) = 15.21, p = 0.01].

Likewise, ANOVA of the normalized data revealed a significant shift in the rhythm following Bright Light + Exercise [interaction: F(15, 150) = 2.61; p < 0.01)], but not following Bright Light Alone or Exercise Alone (Figure 2).

## Discussion

The data suggest that late night bright light elicited a marginally greater circadian phase shifting effect than exercise of the same duration. Moreover, the data suggest that late night bright light followed by early morning exercise had an additive circadian phase-shifting effect.

To our knowledge, this was the first human study to compare phase-shifting effects of bright light and exercise of precisely the same duration; the stimuli were also administered at the same time of day, and at similarly sensitive phase delay regions of the respective PRCs for bright light and exercise. The results suggest that bright light is a stronger zeitgeber than exercise. Nonetheless, the findings that Exercise Alone elicited a mean aMT6s acrophase phase shift that was 84% of that following Bright Light Alone, and that there were similar effect sizes associated with these shifts, contrast with conventional wisdom that bright light is far superior to any other stimulus for shifting the human circadian pacemaker. Yet, the results are consistent with the results from other human studies suggesting comparable phase-shifting effects of bright light and exercise of similar durations [14]. These data suggest that the circadian phase-shifting effect of exercise in humans might be second only to that of bright light.

Although it seems unlikely that low intensity (e.g., <25% maximum capacity) and/or short bouts of exercise (e.g., <15 min) would have analogous effects as demonstrated for dim light and short pulses of light [33,34], these are empirical questions that should be tested. Understanding of the sensitivity of the human circadian system to light has evolved over 50 years involving dozens of studies with progressively greater experimental control. The judgment that bright light is a uniquely potent human zeitgeber might be attributed partly to a relative dearth of research focused on exercise and other zeitgebers.

How exercise compares with bright light as a practical means of shifting the circadian system will require far more research. Undoubtedly, many individuals would find it more practical to use bright light than exercise. On the other hand, bright light treatment is less effective or appropriate for some individuals, and exercise might be preferable to light for shifting the circadian system in some situations, e.g., following transmeridian travel in which it is less convenient to carry a light box. Further dose-response studies manipulating duration and intensity of exercise and light are needed to further address the practical utility of these stimuli.

The optimum option for shifting the circadian system could be found in combining zeitgebers [35]. In our previous research, we found a modest non-significant additive phase-delaying effect of late night/early morning bright light combined with simultaneous exercise (shift of 68 ± 10 min) compared with bright light alone (20 ± 19 min) [36]. Consistent with hamster studies, the present study also suggests an additive effect of bright light and exercise when these stimuli are separated by a few hours. Based on the hamster model, our intent was to separate the bright light and exercise, yet also time these stimuli so that they both occurred at phase-delay regions of their respective PRC. In our previous research using a similar ultra-short sleep-wake schedule, we found that the delay regions of the light and exercise PRCs in young adults were similarly timed, with observable delay shifts beginning about 9 h before the aMT6s acrophase and ending about 1 h after the acrophase. Generally, this timing predicts that phase delays for either light alone or exercise alone are probable from roughly 1900 h to 0500 h.

Post-hoc assessments suggest that within the Bright Light + Exercise treatment, the exercise stimulus fell in the phase-advance region of the exercise PRC for some participants, even if an immediate phase delay following the preceding bright light stimulus is assumed. It seems likely that greater average additive effects might have occurred had the exercise stimulus occurred in the next available wake interval of the ultra-short sleep wake cycle, at 0110–0240, i.e., 3 h after instead of 6 h after the light stimulus. Future research should explore multiple different combinations of timing of bright light and exercise for shifting the circadian system.

The study had several notable limitations. First the sample size was small, though this was partly mitigated by the within-subject design. Second, because the participants were young, healthy, and relatively physically active, the generalizeability of the findings to older individuals and to less active and healthy individuals is unclear. Hamster studies have shown that previously inactive animals show the most dramatic phase-shifting effects of exercise [10,11,12], which could be analogous to greater effects of bright light following adaptation to darkness [37]. Thus, conceivably, less fit/active people would respond equally well to exercise of shorter duration and/or lighter intensity. Third, the generalizability of the data can also be questioned since the exercise stimulus could not be managed by much of the population. Fourth, without a non-treatment control, we cannot make definitive inferences about the extent to which the observed delays are attributable to the experimental stimuli or the drift in circadian phase associated with the ultra-short sleep-wake protocol. Based on previous studies using the ultra-short sleep-wake cycle [27,38], we would have expected the drift between initial and final circadian assessment to be approximately 30 min following each of the treatments.

In summary, the data suggest similar phase-shifting effects of 90 min of bright light and 90 min of exercise, and an additive effect of 90 min of bright light followed by 90 min of exercise. These results contrast with the general opinion that bright light is far superior to other zeitgebers. Further studies are needed comparing dose-response effects of various bright light and exercise durations; examining the effect of these stimuli in the general population or in individuals with circadian rhythms disorders; and examining different combinations of timing of bright light and exercise.

## Competing Interests

The authors declare that they have no competing interests.
